# Factors and processes shaping the population structure and distribution of genetic variation across the species range of the freshwater snail *radix balthica *(Pulmonata, Basommatophora)

**DOI:** 10.1186/1471-2148-11-135

**Published:** 2011-05-20

**Authors:** Markus Pfenninger, Moritz Salinger, Timm Haun, Barbara Feldmeyer

**Affiliations:** 1 Molecular Ecology Group, Biodiversity and Climate Research Centre, Biocampus Siesmayerstraße, Goethe-University, 60323 Frankfurt am Main, Germany

## Abstract

**Background:**

Factors and processes shaping the population structure and spatial distribution of genetic diversity across a species' distribution range are important in determining the range limits. We comprehensively analysed the influence of recurrent and historic factors and processes on the population genetic structure, mating system and the distribution of genetic variability of the pulmonate freshwater snail *Radix balthica*. This analysis was based on microsatellite variation and mitochondrial haplotypes using Generalised Linear Statistical Modelling in a Model Selection framework.

**Results:**

Populations of *R. balthica *were found throughout North-Western Europe with range margins marked either by dispersal barriers or the presence of other *Radix *taxa. Overall, the population structure was characterised by distance independent passive dispersal mainly along a Southwest-Northeast axis, the absence of isolation-by-distance together with rather isolated and genetically depauperated populations compared to the variation present in the entire species due to strong local drift. A recent, climate driven range expansion explained most of the variance in genetic variation, reducing at least temporarily the genetic variability in this area. Other factors such as geographic marginality and dispersal barriers play only a minor role.

**Conclusions:**

To our knowledge, such a population structure has rarely been reported before. It might nevertheless be typical for passively dispersed, patchily distributed taxa (e.g. freshwater invertebrates). The strong local drift implied in such a structure is expected to erode genetic variation at both neutral and coding loci and thus probably diminish evolutionary potential. This study shows that the analysis of multiple factors is crucial for the inference of the processes shaping the distribution of genetic variation throughout species ranges.

## Background

One of the major unsolved questions in evolutionary biology is why the vast majority of species fails to adapt to conditions outside their present niche and, as a consequence, usually exhibit geographically confined range limits [1-5]. Theoretical considerations suggest that local adaptation to conditions outside the current niche depends crucially on the geographic distribution of genetic and demographic characteristics across the species' range [6].

One of the most influential framework on the distribution of genetic variation across species' ranges is the Abundant-Centre Hypothesis (ACH) [7]. It states that individuals of a species should become most abundant in areas where the conditions for reproduction and thus population growth are most favourable. In contrast, the number of populations and population density should decline towards areas with less advantageous environments until survival becomes impossible [4]. Approaching the niche limits, populations should therefore become rarer; less populated and be subject to increased turn-over [2,8-10]. Consequently, geographically marginal populations are expected to harbour less genetic variation and to be more strongly isolated from one another [11], because the population size and its recurrent fluctuations determine the loss rate of genetic variation due to genetic drift.

Asymmetrical gene-flow from larger sized, more abundant central populations to the range margins can counteract the previously described setting. Such gene-flow may prevent local adaptation by constantly supplying 'maladapted' alleles from the core range into marginal populations [6]. Under this scenario, the genetic variation in marginal populations should not differ much from the core area and population differentiation should be low.

A recent exhaustive review across different taxa showed that in about two out of three empirical studies genetic variability indeed decreased and population differentiation increased towards range margins, as expected under the ACH [12]. However, most of these studies were based on rather small parts of the species range or a rather restricted number of populations. Moreover, not only the geographic marginality of a population or its connectivity can influence the genetic variation present. Only few studies so far tested possible alternative factors responsible for the observed patterns and none incorporated a historical perspective. We outline below other factors potentially influencing the distribution of genetic variability across species ranges. Populations may not only be marginal with respect to their geographic position, but also with respect to their environmental habitat quality [5]. Populations inhabiting low quality sites may be subject to increased population turn-over due to challenging environmental conditions and their variability, which may also negatively influence their genetic variability by increased drift [12].

Genetic variability across species ranges may also be influenced by local biotic interactions, in particular by competition with closely related, ecologically similar species or hybridisation with them in parapatric settings [12]. While the former process should result rather in a decrease of genetic variation due to increased population turnover, the latter is predicted to increase genetic diversity due to introgression of alleles in the hybrid zone [13].

Also contingent historic events like presence of geological dispersal barriers, population fragmentations and range expansions e.g. due to Pleistocene glaciations in temperate regions may have exerted their lasting influence on the distribution of genetic variation within a species. Here, the expectations on the distribution depend on the actual population history and may include decrease in genetic variation due to founder effects and population bottlenecks or an increase e.g. in secondary contact areas of previously isolated lineages [10,14-18]. Table 1 summarises the factors expected to influence genetic variability across species ranges, their predicted influence on genetic variation and the population processes by which they act.

**Table 1:** Factors potentially influencing distribution of genetic variation across species ranges, their expected effect on genetic variability and the acting population processes.

Factor	Expected effect on genetic variability in affected populations	Population process
Geographic marginality	negative	Drift by increased population turn-over and low population density because of unfavourable environmental conditions
Gene-flow	positive	Gene-flow counteracts the effects of drift
Mixed mating system	negative	Selfing decreases effective population size and thus increases drift
Environmental marginality	negative	Drift by increased population turn-over and low population density because of unfavourable environmental conditions
Biotic interactions	negative or positive	Drift by increased population turn-over due to interspecific competition Introgression of alleles from related species by hybridisation
Dispersal barriers	positive	Accumulation of immigrating alleles
Range expansions	negative	Drift due to founder effects, bottlenecks or allele surfing
Population fragmentation	negative	Drift due to diminished effective population size
Secondary contact	positive	Mixing of alleles that evolved in isolation

While most factors act on genetic variability in a one-way direction, the mating system both influences genetic variability and its prevalence can be driven by at least some of the above described factors. On the one hand, a mixed mating system decreases the effective population size. Populations with a mixed mating system or purely selfing populations are therefore expected to experience increased drift [19]. Local differences in the proportion of selfing versus outcrossing individuals can thus determine the distribution of genetic variability [20]. On the other hand, habitat stability [21], population density and range expansions [22] can influence the preference for selfing or outcrossing via mating system evolution or phenotypic plasticity.

The factors described above provide alternative, but not necessarily mutually exclusive explanations for the distribution of genetic variation. Thus testing only a single factor at a time may lead to erroneous conclusions on the factors and processes governing the distribution of genetic variability over species' ranges [12]. Empirical studies explicitly addressing these hypotheses comprehensively are therefore needed to understand these factors and processes more fully [12].

In the present study, we tackled this issue using a pulmonate freshwater snail *Radix balthica *as model organism. This species is one of several species in the morphologically cryptic species complex *Radix *Montfort 1810 [22]. It is distributed throughout North-Western Europe from Northern Sweden to the South of France over a wide range of environmental conditions. As in many other pulmonate species [23,24], *R. balthica *is suspected to have a mixed mating system [19]. Without demanding a particular substrate or water quality, the species occurs in rather lentic water bodies like the shore zone of lowland lakes and ponds, oxbows, irrigation channels and fountains, but also in slow flowing rivers and streams [25,26]. Like in most other non-flying freshwater organisms, active dispersal depends crucially on continuous habitat; however, water-fowl mediated passive transport is probably the major mechanism for dispersal among unconnected habitats [27,28]. With the mentioned characteristics, the species is typical in most regards for many freshwater molluscs and other freshwater invertebrates lacking active long range dispersal capacities.

Since more than a single factor may contribute to the distribution of genetic variability, we analysed the population structure, mating system and simultaneously tested the influence of the various factors outlined above by assessing the geographic distribution of supposedly neutral nuclear and mitochondrial genetic variability across the species range of *R. balthica *using statistical modelling and model selection techniques.

## Results

### Sampling

**Figure 1:**
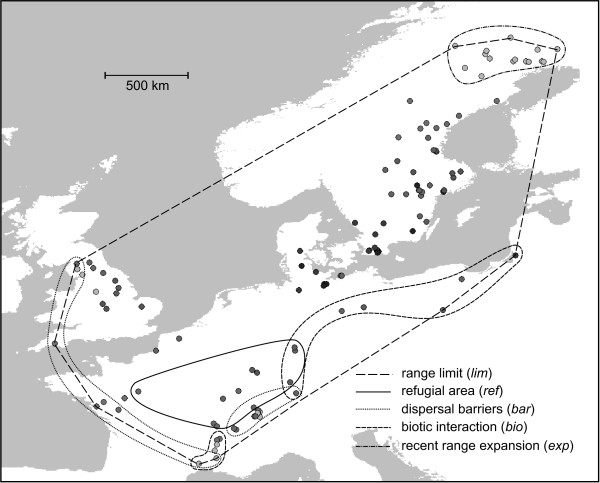
**Sampling site distribution and their grouping to predictor variables**. Circles represent sampling points. The colour gradient from light grey (extreme climate) to black (average climate) represents environmental marginality (marg) regarding climate variation as inferred from PCA analysis (see Additional File 3). The convex polygon around all sampling points indicates the species range limits considered. Populations grouped to different predictors are indicated by differentially hatched lines. The Holocene expansion area (*hol*) comprises populations neither situated in the refugial nor in the recent range expansion area.

We identified 1084 individuals sampled from 64 sites as *R. balthica *with DNA barcoding. Together with previously identified *R. balthica *populations, this resulted in the first comprehensive molecularly confirmed estimate of the species range (Figure 1). In total, more than 150 sites with *Radix *specimen were barcoded. For their spatial distribution and the distribution of other *Radix *taxa, see Additional File 1.

Including samples from previous studies, we genotyped 1457 individuals from 81 sampling sites with eight microsatellite markers. For seven sites used for microsatellite analysis, less than ten individuals could be typed, leading to an unbalanced sampling. However, since omitting these sites from subsequent analyses did not change the results, we did not exclude them from the study. COI sequence data of more than 400 bp length was analysed from 798 individuals sampled at 66 sites (GenBank accession numbers of new sequences HQ244502-HQ244993, GU735965-GU736200, other sequences used were from [22] and [29]).

### Population genetic structure

**Figure 2:**
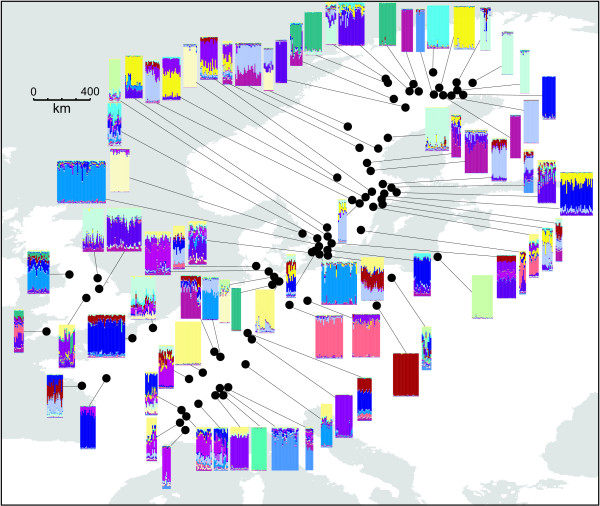
**Population structure analysis inferred from Bayesian clustering**. Each bar corresponds to the cluster membership proportions (k = 20) of an individual as estimated from microsatellite data. The more colours appear in a bar, the more admixed is the individual. The bars from a sampling site are arranged in blocks, connected with a line to the respective sampling site. Populations with similar genetic composition have therefore blocks with similar colour patterns.

**Figure 3:**
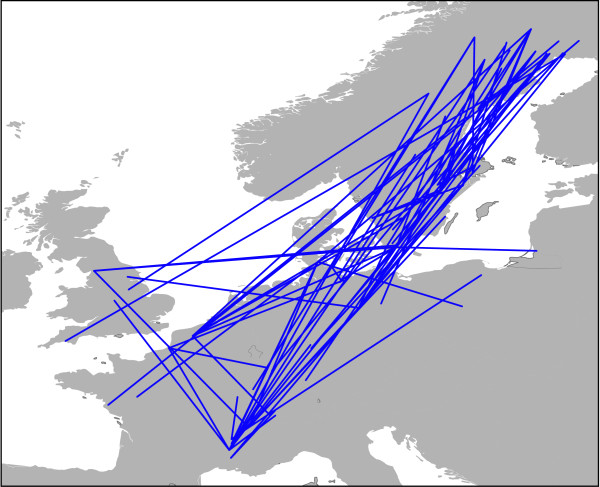
**Plot of minimum spanning tree on distribution map**. Based on their nuclear differentiation most similar populations are connected by a blue line. Clearly, populations along a Southwest-Northeast axis are clustered together.

**Figure 4:**
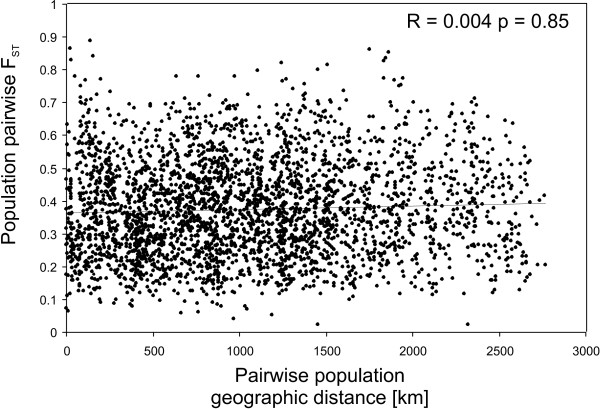
**Plot of pair-wise geographic population distances against the population pairwise linearised F_ST _estimated from microsatellite data**. The null hypothesis of no correlation could not be rejected *r *= 0.04, *p *= 0.84.

The average overall *F*_ST _estimate was 0.368 +/- 0.400 (mean +/- *s.d*.). The Bayesian cluster analysis indicated that the hypothesis of 20 clusters was most strongly supported by the data (LnD = -28,578; *s.d*. = 209). The colour coded cluster memberships of each individual are depicted in Figure 2. There was no obvious geographical pattern; many sampling sites harboured individuals with a single majority cluster membership, but there were also sites with highly admixed individuals. Also the distribution of the clusters followed no obvious pattern; sites with different clusters were found in close proximity while the same clusters were found hundreds of kilometres apart (Figure 2). The minimum population spanning tree revealed, that the most similar populations were, with few exceptions, arranged in Southwest-Northeast direction, however, regardless of geographic distance between them (Figure 3). This was also reflected in the plot of population pairwise *F*_ST _s against the geographical distance (Figure 4). The null hypothesis of no influence of geographical distance on genetic similarity could not be rejected (*r *= 0.004, *p *= 0.85). It was thus not necessary to correct the following analyses for geographical distance among populations [30].

### Genetic diversity and mating system estimates

The average expected heterozygosity over all loci (*H_E_*) was 0.448 +/- 0.168 (mean +/- *s.d*.) with an observed minimum of 0.068 (sampling site SKJ) and maximum of 0.955 (FTO). The average number of alleles per locus (*A*) was 15.6, the overall rarefied value per sampling site and locus 3.207 +/- 1.312. The observed minimum value was 1.370 (SKJ), the maximum 7.734 (SSO). The correlation between *H_E _*and *A *was very high (r = 0.85, p < 0.001).

At least partial selfing was inferred for 47 out of 81 populations (58%). The average population selfing rate was 0.20 +/- 0.25. The maximum value observed was a completely selfing population (s = 1.00, SSK). The estimated degree of self-fertilisation was only poorly correlated to the genetic variability measures *H_E _*and *A *(r = 0.30, p = 0.006 and r = 0.22, p = 0.045, respectively).

A total of 132 mitochondrial haplotypes was identified over the species range. After rarefaction, 4.085 +/- 2.193 haplotypes per sampling site were observed, ranging from a single haplotype (ALL, LJO, SHU) up to 11.765 different haplotypes (SKR).

All measures of diversity per sampling site and a graphical representation of their spatial distribution can be found in Additional File 2.

### Inference of population bottlenecks

It was possible to test the 34 non-selfing populations on signs of recent population bottlenecks. Nine (26%) of these showed a significant heterozygous excess. The populations with recent bottlenecks were widely distributed over the species range, but not in the recent expansion area (see Additional File 2 Figure A4).

### Effects of single predictors on genetic diversity and mating system

**Figure 5:**
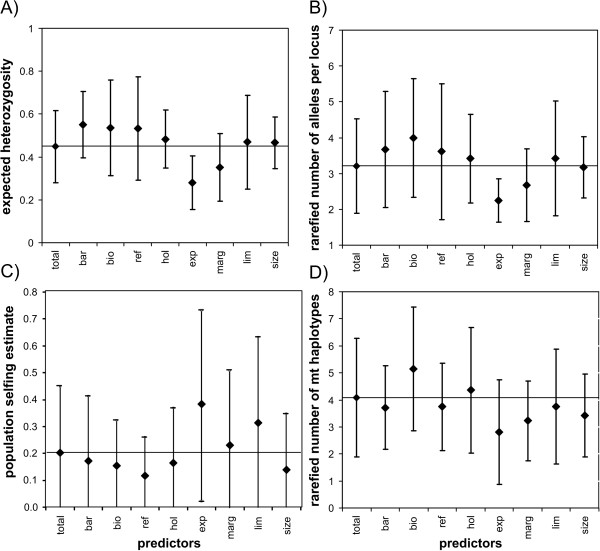
**Plot of the mean (+/- s.d.) of the genetic and selfing estimate measures for populations grouped according to predictor variables**. The overall mean = total (+/- *s.d*.) of the respective measure is given as comparison. A) expected heterozygosity (*H_E_*), B) number of rarefied alleles (*A*), C) population selfing estimate (*s*)and D) number of rarefied haplotypes (*H_mt_*). For the dichotomous variable *size*, the mean for the smaller habitats are presented.

Expected heterozygosity (*H_E_*) was above the overall average in the sampling sites grouped by the predictor variables dispersal barrier (*bar*), biotic interaction (*bio*), LGM refugia (*ref*), Holocene expansion (*hol*) and distance to range limit (*lim*). By contrast, it was reduced relative to the mean in the expansion sampling sites (*exp*) and environmentally marginal sites (*marg*, Figure 5A) This pattern was identical for the rarefied average number of alleles per locus (Figure 5B).

The population selfing estimate (*s*) was on average lower than the overall average in sites grouped by the predictor variables *bar*, *bio*, *ref*, *hol *and *size*, while it was higher in *exp*, *marg *and *lim*. However, the variance was very high in each group (Figure 5C).

The number of mitochondrial haplotypes (*H_mt_*) was increased at sites with presumed biotic interaction (*bio*) and to a lesser extent in the Holocene expansion sites (*hol*). In all other groupings, the haplotype diversity was decreased with the strongest effect observed in the recent expansion sites (*exp*, Figure 5D).

As the difference in all diversity measures from the refugial area and the Holocene expansion sites were not significantly different from zero, these categories were merged and contrasted against the effect of the recent expansion area in subsequent analyses.

### Selecting among models explaining the distribution of diversity

Almost all models explained a portion of variance significantly larger than zero at the 5% error probability level or less. The models highlighted below were all highly significant (*p *< 0.0001).

The distribution of expected heterozygosity (*H_E_*) was explained by the additive effect of four models with two or three variables. It was best supported by the additive effect of dispersal barriers (*bar*) and expansion area (*exp*) (Akaike weight 0.56; see Table 2). In all models, *exp *explained by far most of the variability (> 68%).

**Table 2:** Predictor combinations in statistical modelling with more than 5% support in Akaike weights

*Measure of diversity */Factor combinations		d.f.	SS_res_.	% explained variance	AIC	Akaike weight
*H_E_*	N = 80					
bar+exp		2	1.676	92.5	-76.22	0.56
bio+exp		2	1.685	92.4	-75.79	0.24
bio+exp+marg		3	1.654	92.6	-75.28	0.09
bar+exp+lim		3	1.660	92.6	-74.99	0.05
*A *	N = 80					
bio+exp		2	115.06	90.1	262.10	0.88
bio+exp+marg		3	113.87	90.2	340.09	0.09
*H_mt_*	N = 66					
bar+exp+lim		3	273.23	79.9	289.06	0.95
*s*	N = 80					
marg+exp+lim+size		5	4.06	49.0	122.1	0.97

All possible predictor combinations were tested with Generalised Linear Modelling and the support of all models by the data inferred by the Akaike Information Criterion (AIC). Akaike weights were calculated on the basis of all models tested. *H_E _*= expected heterozygosity, *A *average number of rarefied alleles per locus, *H_mt _*number of rarefied mitochondrial haplotypes, *s *estimate of population selfing rate.

The rarefied number of alleles *A *was best explained by the additive effect of the factors biotic interaction (*bio*) and *exp *(Akaike weight 0.88), followed by *bar*, *exp *and environmental marginality (*marg*) (Akaike weight 0.09, Table 2). Also here, *exp *accounted for most of the explained variance (79%).

Variance in population selfing estimates was best explained by the additive effects of the four variables *marg*, *exp*, *lim *and *size *(Akaike weight 0.97). However, only 49% of the total variance was explained by this model (Table 2).

The haplotype variability was best explained by the additive effect of the model with three variables *bar*, *exp *and range limits (*lim*) (Akaike weight 0.95, Table 2). The contribution of *exp *dominated the explained variance (79%).

### Degree of differentiation among classes of populations

**Figure 6:**
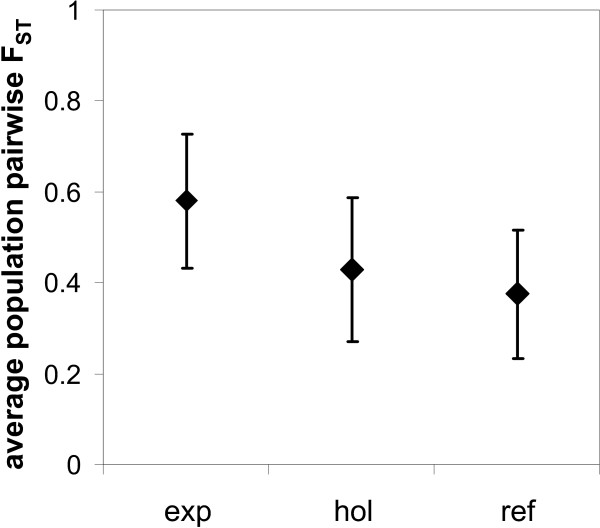
**Test on heterogeneity in population differentiation among central vs. marginal populations**. Shown are the mean *F*_ST _(+/- *s.d*.) in the recent expansion area (*exp*), the Holocene expansion area (*hol*) and the LGM refugia (*ref*). There is a significantly stronger structure in the expansion area than in both other areas (1000 simulations, *p *< 0.001 for both comparisons). The comparison between *hol *and *ref *was not significantly different.

Except for recent expansion (*exp*), none of the other predictors yielded a significantly stronger or weaker structured grouping. In the recent expansion area, the average population pair-wise *F*_ST _was 0.198 units higher than in the remaining range. This difference proved to be significantly different from zero with an error probability of less than 0.001 according to the randomisation test employed (1000 permutations per test, Figure 6).

## Discussion

### Population structure is dominated by passive dispersal

Like in most flightless freshwater taxa, dispersal of *R. balthica *between unconnected habitat patches depends on passive dispersal mechanisms [31]. In particular lentic habitats are ephemeral on an intermediate time-scale, thus selecting on populations with good dispersal capacities [32]. In *R. balthica*, this passive dispersal mechanism is presumably transportation by water fowl [33]. The minimum spanning tree (Figure 3) adds credibility to this assumption, as it clusters the respectively most similar populations mainly along the major bird migration route of the East Atlantic flyway in Southwest-Northeast direction. The suggested connection pattern of the minimum spanning tree beard a striking resemblance to the inferred initial postglacial recolonisation dispersal pattern, where also bird migration routes were implicated [29]. This suggests that it either presents the remnant of this saltatory postglacial colonisation process or that recurrent dispersal follows the same routes. The connection lines of the respectively most similar populations appeared to be distance independent (Figure 2). This was also reflected in the spatial distribution of the inferred genotype clusters (Figure 2), where similar genotypes could be found hundreds of kilometres apart and/or in close proximity. Both findings are substantiated by the complete lack of correlation between population differentiation and geographic distance (Figure 4). Thus, distance independent passive transport seemed to be the primary process for gene-flow and/or colonisation of empty habitats along the Southwest-Northeast axis from virtually any part of the environmental gradient to any other. As a consequence of this unpredictable long range dispersal, colonisers originating from one part of the range must cope with very different environmental conditions upon arrival, arguing for a high phenotypic plasticity leading to the observed broad ecological tolerance.

Despite the possibility for virtually unrestricted long range dispersal, only few populations were found to show admixture; most sites harboured primarily individuals that clearly belonged to the same inferred genotype cluster (Figure 2). This matched the observation that despite the large overall number of alleles per locus (15.6) and haplotypes (132), at single sites, only a very limited number of haplotypes was found (3.2 +/- 1.3 alleles per locus and 4.2 +/- 2.1 haplotypes, respectively). Such a pattern is compatible with a scenario of site colonisation by one or few individuals, followed by a rapid increase of the population size, supported by the inferred mixed mating system in *R. balthica*. The widespread presence of selfing supplements thus the finding of a preferentially outcrossing system in a local flood-plain system of the Rhône river by Evanno et al. [34].

Indeed, the mating system, in particular the ability to reproduce uniparentally has long been considered to influence colonisation success [35]. Selfing, like any form of uniparental reproduction, has the automatic advantage of increased gene-transmission to the next generation (no cost of sex), thought to be balanced by the costs of inbreeding depression [36]. Selfing can evolve as reproductive assurance strategy in the absence of mating partners, because it is always better to self-fertilise offspring whose fitness may suffer from inbreeding than to leave no offspring at all [37]. Predominant selfing as mating system should therefore evolve mostly in cases where mating partners are rare or absent [21], which is in particular the case for the first colonisers of a previously empty habitat. Even Darwin [35] suggested that selfing or monoecious plants should expand their ranges more easily because already a single individual can found a reproducing population. Indeed, the average proportion of selfing was slightly increased in the recent expansion area (Figure 5C). Such populations, made up of selfing and/or inbred individuals, would be relatively inert against the effect of subsequent gene-flow, as the establishment probability of immigrating alleles in a demographically large population is low [38]. Another, not mutually exclusive explanation for the observed pattern would be short population persistence times, not allowing to accumulate genetic variation by gene-flow or mutation over time. Other studies on freshwater snails have shown that high population turn-over and large size fluctuations are indeed typical for this taxon in general [39-42] and for *R. balthica *in particular [43]. The bottleneck analysis with the non-selfing populations indicates that the population dynamics of the species is indeed high and not restricted to certain parts of the species range. Nine out of 34 populations (26%) tested showed signs of a population bottleneck within the last few generations (see Additional file 2 Fig. A4).

The observed pattern could also point to a low incidence of successful dispersal events, resulting in low gene-flow rates. This is, however, difficult to evaluate, because direct estimates of passive dispersal rates are not available for freshwater snails.

### Current climate change left its mark in the distribution of genetic variability

The influence of the various predictors on all measures of genetic variability was remarkably similar in terms of direction of deviation from the overall mean (Figure 5). This confirmed that both nuclear and mitochondrial markers were subject to similar demographic forces, as might be expected in simultaneous hermaphroditic animals where e.g. sex biased dispersal or sex ratio bias are by definition impossible. The effects on the number of rarefied microsatellite alleles per locus *A *and expected heterozygosity *H_E_*were so similar (correlation coefficient r = 0.85) that we will discuss them together hereafter (Figure 5). Even though selfing proved to be a substantial issue in *R. balthica*, the mating system population differences had a surprisingly low effect on the distribution of genetic variability, as shown by the low correlation between the degree of selfing and genetic variability measures *H_E_*and *A *(r = 0.30, p = 0.006 and r = 0.22, p = 0.045, respectively). This means that high selfing rates are not predominantly responsible for the loss of genetic variability. A low correlation further allowed investigating whether the factors considered influenced the mating system. The predominant factor in all models with substantial support was the recent, climate driven range expansion (*exp*), which lowered the level of variability for all genetic markers considerably (Figure 5). This is not surprising, as an ongoing or recent expansion represents a non-equilibrium situation caused by repeated bottlenecks and founder events both of which decrease genetic variability [44,45]. In *R. balthica*, this effect might be enhanced by the possibility of self-fertilisation [46], which facilitates the colonisation of newly emerging habitats by one or few individuals [47-49]. The factor *exp *was part of the best model to explain the distribution of selfing, showing that this trait may have played a role in the swift colonisation of newly emerging habitat in the course of a climate change. 'However, given enough time, one may predict that the effects of this non-recurrent, historic event at the current range limit will be transient and eventually assume a level of genetic variation either by immigration or mutation comparable to the remaining distribution area. In the past, this has obviously been the case for the expansion from the Pleistocene refugia into the Holocene expansion areas, where nowadays no appreciable difference in genetic variability was detectable (Figure 5).

Biotic interactions had a positive effect on the intra-population variability of both nuclear and mitochondrial markers (Figure 5A, B, Table. 2), however in the GLM analysis only on *A *and *H_mt_*. According to Eckert *et al*. [12], such an increase may be explained by introgression from neighbouring, closely related species through inter-specific hybridisation. However, close inspection of the alleles and mitochondrial haplotypes found at the sampling sites in question revealed, with the exception of one private allele and one private haplotype in one population, respectively, solely alleles and haplotypes that also occurred in other *R. balthica *populations throughout the species' range. Moreover, the allelic size range of the microsatellite loci in the potentially hybridising undescribed *Radix *species is known [46] and none of these alleles were found in the present data set. Also the mitochondrial haplotypes found at these sites fit very well in the haplotype variability of *R. balthica *[29]. Inter-specific hybridisation with neighbouring taxa is thus an unlikely explanation for the pattern reported here.

However, secondary contact of two more *R. balthica *lineages, *e.g*. from different refugial populations, could be the reason for the increase of genetic variability in these areas, as has been shown for other snail species [50,51]. Several sites throughout the range show signs of increased nuclear admixture, in particular in Southern Sweden and around the LGM refugia (Figure 2, 5). Since most sites grouped in the variables *bio *are situated around the refugial area and overlap in these more than average variable populations with the predictor *bar *(Figure 1), an increased variability of nuclear and mitochondrial markers predicted by these variables may indeed be due to few admixed, secondary contact sites and not due to the biological process tested for.

Population size, as rather crudely estimated from the size of the water body, had no detectable effect on the distribution of genetic variability (Figure 5, Table 2). This may have two major reasons: first, population densities of more than 50 individuals per m^2 ^were observed and thus population sizes of several thousand individuals even in small water bodies can be reached (personal observation M. Pfenninger). Thus, the effect of drift in small populations may be difficult to estimate from habitat size alone, but depend rather on the mating system or the founding history. Second, freshwater snail populations are often subject to high population turnover or size fluctuations [21,52] which lead to a discrepancy between the demographic and the effective population size and thus, potential loss of genetic variability. The high proportion of bottleneck populations detected, argue in that direction.

The size of a water body, however, did have an effect on the selfing rate (Figure 5C, Table 2). Surprisingly, larger habitats were associated with more selfing. This is perhaps due to a dilution effect in larger habitats, which makes selfing as a reproductive assurance strategy more often necessary, because potential mates are less often encountered.

Loss of variability by extinction-recolonisation dynamics was also substantiated by some of the models incorporating environmental marginality (*lim*) that received substantial support in the data (Table 1). Sites facing more extreme environmental variation exhibited a slightly decreased level of genetic variability at nuclear markers (Figure 5). This is probably a result of extreme climatic events, like e.g. droughts too severe for the snails, flash-floods or too cold winters, in these areas. Such events are expected to decrease genetic variation by decimating or extinguishing local populations and have been shown for *R. balthica *on a local scale [43].

Geographic marginality *per se *contributed little to the distribution of genetic variability in *R. balthica *(Table 1). In nuclear marker loci, populations close to the inferred range limits even tended to harbour slightly more genetic variation than the total average (Figure 5). Contrary to the majority of empirical studies reviewed by Eckert *et al*. [12], the distribution of genetic variability in *R. balthica *does not follow the predictions for the genetic extension of the ACH. However, contrary to all previously discussed factors, the reliability of this inference depends crucially on the quality of the inference and sampling of the range and its margins. Apart from the multitude of possible definitions for a species range [53], its practical determination is inherently difficult, because it depends as well on the presence of unequivocally identified populations of the focal species in certain areas as on their absence in others. While the former often enough presents a practical problem due to unrecognised cryptic species[54], varying observation density and -quality [55], it is virtually impossible to prove the absence of most species from an area. A species range and in particular its margin is therefore rather an effort-dependent estimate than a fact.

In the case of *Radix*, unequivocal species determination is possible only with molecular methods and in particular *R. balthica *can be easily mistaken for other species [19]. Therefore, range estimates of *R. balthica *based on morphology or even anatomy are prone to error and were not considered here. Our estimate of the R. balthica range represents therefore the best currently available estimate. However, given the postglacial expansion history as inferred by phylogeography [33], it cannot be excluded that the species also occurs in Norway, Ireland and Scotland. On the other hand, the absence of *R. balthica *and the confirmed presence of other MOTUs in the sites sampled in the South-West, South, South-East and East argues for a good coverage of the range limits in this area (see Additional file 1). For the South-East, the absence of *R. balthica *from the Balkans is confirmed by another recent study [56]. In Sweden, no *Radix *snails were found further North than the populations reported here during our surveys. In total, we are confident that our sample represents i) the larger part of the present species range and ii) that with the possible exception of the North-West, also the range margins were adequately sampled.

However, the ACH does not predict precisely, how variation should decrease towards range margins [12]. By testing the distance to the closest range margin, we assumed that the decline is steady and linear from the core range. If the decline is actually steep and starts only close to the margins, we would have missed it with our sample strategy, because we have probably missed the respectively most marginal populations. On the other hand, a range margin effect requires distance-dependent dispersal [12], which we have shown to be absent in this species.

The factors evaluated here had also an impact on the variability in the mating system. The common quality of the factors identified to trigger changes in mating system towards more self-fertilisation seemed to be increased population turn-over (Table 1). Actually, self-fertilisation should be advantageous in any metapopulation system with high population turn-over rates [57]. However, even the best model (*marg *+ *exp *+ *lim *+ *size*) explained not even half of the variance in selfing, indicating that probably additional, untested factors significantly shaped the mating system.

## Conclusions

The process mainly responsible for the population structure and distribution of genetic variability measured as nuclear and mitochondrial across the species range of *R. balthica *was found to be passive, probably bird-mediated, distance independent dispersal along a Southwest to Northeast axis. Apart from the expected effects of a recent range expansion, other processes or factors suggested in the literature had only a minor effect on the geographic distribution of genetic variability. This dispersal mode led to high overall genetic variability, but locally impoverished populations. Low local variability, along with high population turn-over (particularly in climatically marginal populations) and range-wide dispersal dynamics argue against a high evolutionary potential [6,58], This is because the increased local demographic drift acts on all parts of the genome, thus eroding not only the neutral variation but also standing genetic variation at coding or regulatory loci. It is thus likely that the observed population structure prevents local adaptation unless very strong selective forces counteract the strong drift [6]. A recent study could not detect local adaptation in *R. balthica *on a regional scale [34]. However, this supposition needs confirmation by ecological and physiological experiments for populations from the entire species range.

The particular population structure observed is thus probably the main explanation for the previously inferred intraspecific climatic niche conservatism from the LGM to the present day [29]. It is likely that the wide physiological tolerance to the array of conditions encountered in the current species range, finally also limits the distribution of *R. balthica*.

As in particular species inhabiting ephemeral habitats, (e.g. lentic freshwater, wood glades) show similar population structures and dispersal dynamics [32], the conclusions from the present study concerning expected niche conservatism may therefore also apply to many other taxa with similar characteristics.

## Methods

### Range sampling and *Radix *taxonomy

*Radix *populations were sampled in the presumed range throughout North-Western Europe. Because taxonomic identity cannot be deduced from morphologic features in this genus, all individuals used in this study were DNA barcoded, for which a COI sequence of less than 300 bp proved sufficient for unequivocal species identification [22]. In this study at least five reproductively isolated molecularly defined operational taxonomic units (MOTU) were found. MOTU2 was one of two lineages present in Sweden and, together with the fact that it is statistically associated with leaner shells than the other Swedish lineage (MOTU4, *R. auricularia*[22]), therefore best fits the description and locus typicus ("*habitat ad M Balthici littera"*) of *Radix balthica *(L., 1758). We associated therefore the biological entity MOTU2 with the taxonomic name *R. balthica *and will use this name hereafter.

### Site sampling

To avoid potential Wahlund effects, individuals were sampled along a shoreline of max. 12 m, which is well within the estimated neighbourhood area of *R. balthica *(approx. 125 m shoreline, M. Salinger, unpublished data). Additionally, we used stored DNA and published data, respectively, from individuals sampled for previous studies [22,33,46].

### Microsatellite and mitochondrial haplotype analysis

DNA was extracted using glass fibre DNA extraction after a protocol developed by the Canadian Centre of DNA Barcoding [59]. Cytochrome oxidase subunit I (COI) fragments were amplified using PCR, performed with Invitrogen Taq DNA polymerase and universal primers published by Folmer *et al*. [60]. Sequencing reaction was performed using ABI Prism Big Dye terminator kit (Perkin-Elmer). Sequenced fragments were separated and read on an ABI Prism 3730 capillary sequencer (Applied Biosystems).

All snails were genotyped at eight highly polymorphic microsatellite loci [46]. Multiplex microsatellite amplification was carried out using QIAGEN Type-it™ microsatellite PCR Kit with fluorescent dye labelled forward primers [46]. PCR products were separated using an ABI Prism 3730 capillary sequencer (Applied Biosystems) with GeneScan™ 500-LIZ™ size standard (Applied Biosystems). Microsatellite allele lengths were analysed using GENEMAPPER 4.0 software (Applied Biosystems).

### Population genetic structure

The population genetic structure was estimated by population pair-wise *F*_ST _s and the overall *F*_ST _for both nuclear and mitochondrial markers, calculated in Arlequin 3.1 [61]. Additionally, the assignment of all individuals to genotype clusters was performed with the software STRUCTURE[62]. STRUCTURE implements the Markov Chain Monte Carlo (MCMC) algorithm for the generalized Bayesian clustering method to classify individuals into clusters using genotypic data of unlinked markers. We used the *location prior *option, implementing the assumption that individuals from the same location have a higher probability to stem from the same population than individuals sampled at different locations. A uniform prior for alpha was applied for all populations, with an initial value of 1. We used the admixture model assuming a number of clusters from *K *= 1 to *K = *40. All MCMC runs were repeated five times for each value of *K *for 200,000 generations with 25,000 burn-in steps. We used the maximum LnP(D) value to infer the most likely number of clusters, given the data. LnP(D) is the log likelihood of the observed genotype distribution in *K *clusters. The analysis was repeated also without location prior, as recommended by Falush *et al*. [63]. To visualise the relations among the populations and main directions of past or present gene-flow, we calculated a minimum spanning tree from a linearised F_ST _matrix using NTSYSpc version 2.0 and plotted it on a map. Populations pair-wise linearised *F*_ST _s were calculated in Arlequin 3.1 [61].

Spatial autocorrelation between populations may bias tests for difference in genetic distance and genetic variation [30]. To test whether geographically closer populations also tend to be genetically more similar in *R. balthica*, we plotted the population pair-wise linearised *F*_ST _estimates for the microsatellite data against the geographic distances among the respective populations. Statistical significance was tested with the Mantel's test option in Arlequin 3.1 [61] with 10,000 permutations.

### Estimates of genetic diversity

For each sampling site with at least seven genotyped individuals, we calculated two different measures of within-population nuclear diversity: arcsin transformed expected heterozygosity (*H_E_*) and allelic richness (*A*), expressed as average number of alleles per locus. Although both measures are interrelated, *A *is more affected by stochastic drift than *H_E _*and therefore the more sensitive measure [64]. We computed *H_E _*for each population using ARLEQUIN 3.1 [61]. The average number of alleles per locus and population was extracted from the raw data using GENALEX[65]. As the latter measure is sensitive to differences in sample size [12], we rarefied *A *to the minimum number of individuals sampled per site, applying a method of rarefaction recommended for standardisation of allelic richness [66,67].

Within population diversity in the mitochondrial genome was estimated from the number of COI haplotypes (*H_mt_*). To obtain more precise estimates, only individuals for which more than 400 bp congruent sequence information was available were included. The number of haplotypes per population was extracted from the data using DNASP[68] and also rarefied to the minimum sample number to account for different sample sizes.

### Estimation of population selfing rates

The selfing rate was estimated with the g2 estimator using the program RMES [69]. Since RMES is based on multilocus second order heterozygosity disequilibrium in populations and thus independent of the estimation of allele frequencies, the program skirts two major sources of error in calculating the selfing rate: The appearance of null alleles and partial dominance of alleles which can both elevate homozygosity estimates and thus bias selfing estimates based on heterozygous deficiency F_IS_.

### Inference of population bottleneck events

We used the Wilcoxon signed-ranks test implemented in the program BOTTLENECK [70] to detect recent population bottlenecks. This test is based on the assumption that populations having experienced a recent reduction in effective population size exhibit a more rapid reduction of allelic diversity than heterozygosity (i.e. gene diversity) at polymorphic loci; the population is thus not in mutation-drift equilibrium. To detect if the observed heterozygosity is increased in comparison to the heterozygosity expected from the number of alleles in a population, we used the two-phased model of mutation (TPM), which is most appropriate for our microsatellite data set consisting of mostly one-step mutations and a small percentage of multi-step changes [71]. Because a mixed mating system may also influence the mutation-drift equilibrium and may thus bias the estimates of bottlenecks [70], we performed the analysis only for populations with an estimated selfing rate of zero.

### Factors potentially shaping genetic diversity

#### Geographical marginality

To test whether the geographic position of a population in relation to the putative range limit negatively influences genetic variability as expected by ACH, we measured the nearest distance of each population to the putative range margin as a continuous estimator of geographic marginality (*lim*) (Figure 1). Assuming that the sampled populations are a good representation of the range, we constructed a smallest-enclosing-polygon around all sampled populations as a surrogate for the range margin.

#### Environmental marginality

To assess the environmental marginality, we extracted 35 climatic parameters (e.g. precipitation, various temperature and Bioclim parameters) for each sampling point for the period from 1960 - 2000 from publicly available WorldClim data, incorporated in DIVA-GIS [72]. We used a principle component analysis based on a correlation matrix (PCA, Additional File 3) to reduce the strong co-linearity within these data. A continuous estimator of environmental marginality (*marg*) was then gained by calculating the Euclidian distance of each population from the origin of the coordinate system spanned by the significant PCA axes. To infer visually whether this and the previous predictor *lim *tended to result in higher or lower than average diversity measures, sample sites falling within the 85% quantile were contrasted to the remaining populations.

#### Habitat size

The factor determining genetic drift experienced by a population is the effective population size [64]. As direct estimates for population size at the sampling sites were not available, we used habitat size as proxy. We grouped sampling sites in two categories (dichotomous categorical predictor *size*): small = drainage ditches, small streams and ponds smaller than approximately one hectare; large = lakes larger than one hectare. Besides the obvious influence of habitat size on the population size, the extension of a water body may also influence the probability to be the target of long range passive dispersal and thus of successful gene-flow, positively affecting genetic diversity. Additionally, larger water bodies are expected to be more stable than smaller ones, allowing potentially accumulation of genetic variation over a longer time period.

#### Potential biotic interactions

Biotic interactions may play a role in *R. balthica *in the eastern and south-western parts of the range. Here, predictive niche modelling suggested larger suitable ranges than are actually occupied by *R. balthica *[29]. These areas, however, are occupied by other *Radix *species as identified by barcoding [22] (Additional File 1). We have therefore contrasted populations bordering predicted suitable habitat inhabited by congeneric species to obtain a two-categorical predictor of potential biological interactions (*bio*).

#### Range expansions

Based on the results from [29], we contrasted populations in inferred refugia (*ref*), potentially retaining the larger part of the genetic diversity [73], against the species range attained during the Holocene (*hol*, Figure 5). To check whether *R. balthica *has already expanded its range as a consequence of ongoing climate change as suggested for freshwater benthos [74], we analysed the publicly available long term database of the Swedish University of Agricultural Sciences (SLU) (available at http://www.ma.slu.se) with canonical correspondence analysis (see Additional File 4). According to this analysis, *R. balthica *has expanded its range northwards from about 1995 on by at least 200 km as a consequence of increased lake temperatures. This allowed additionally distinguishing these only recently colonised populations from the earlier Holocene expansion and the refugia in a separate predictor (*exp*).

#### Dispersal barriers

As the range of *R. balthica *is limited by the Alps, the Atlantic Ocean and the Mediterranean, we considered populations bordering these dispersal impeding structures in a categorical predictor (*bar*). The geographical distribution of the sampling sites and their grouping in predictor variables is illustrated in Figure 1.

### Statistical modelling

Statistical modelling was employed to evaluate the relative influence of the predictors described above on all measures of population diversity (*H_E_*, *A*, *s*, *H_mt_*) in turn. We used all possible predictor combinations in a Generalised Linear Model (GLM) approach to compute the residual sums of squares (SSR) in the software package STATISTICA[75]. As the natural experiment situation did not allow for a full factorial design, not all interactions could be estimated. From the SSR and the respective degrees of freedom (*d.f*.), the Akaike information criterion with correction for small sample size was then computed for each model [76]. Relative explanatory power of the models was explored by calculating Akaike weights.

### Degree of genetic differentiation among classes of populations

Declining population size and gene-flow among populations towards range margins should also result in increased differentiation among populations [12]. For all categorical classification schemes (*lim, exp, marg *etc.), we have therefore compared the average among population differentiation in this category with the differentiation among the respectively remaining populations. Because pairwise *F*_ST _values are not independent data points, we have applied a simple randomisation scheme to assess the statistical significance of the observed differences. To test whether the observed average *F*_ST _difference between the populations in the respective category and the rest was larger than expected by chance, we created a null distribution by randomly shuffling populations 1000 time among the contrasted categories.

## Authors' contributions

MP conceived the study, analysed the data and drafted the manuscript, MS sampled most of the sites, performed parts of the molecular work, the initial analyses and contributed to drafting the manuscript, TH performed parts of the molecular work and commented on the manuscript, BF contributed to writing the manuscript.

## Acknowledgements

We thank Martin Plath, Mathilde Cordellier, Eugenia Zarza, Aline Depráz and three anonymous reviewers for constructive comments on a previous manuscript version. The work received financial support within the AQUASHIFT priority programme from the DFG (grant MP390/4-2) and was supported by the research funding programme "LOEWE - Landes-Offensive zur Entwicklung Wissenschaftlich-ökonomischer Exzellenz" of Hessen's Ministry of Higher Education, Research, and the Arts.

## Supplementary Material

Additional file 1 **Distribution of *Radix *taxa**. Spatial distribution of the *Radix *MOTU as defined in Pfenninger et al. 2006 plus an additional, newly discovered taxon. This map is the basis for the inference of the species range of *R. balthica*. Click here for file

Additional file 2 **Sampling site table and spatial distribution of diversity indices, selfing estimates and inferred population bottlenecks for *R. balthica***. Table of sampling site code, geographical position in decimal degrees latitude and longitude, number of individuals analysed with microsatellites (N_nuc_), expected heterozygosity (H_E_) and standard deviation across loci, mean rarefied number of alleles per microsatellite locus (A) and their standard deviation, number of individuals analysed for mitochondrial variation (N_mt_), rarefied number of mitochondrial COI haplotypes (H_mt_), number of individuals measured for body size (N_size_). Figures A1 - A3 show a graphical representation of the spatial distribution of H_e_, H_mt _and, s, respectively. Click here for file

Additional file 3 **Assessment of environmental marginality**. PCA (principle component analysis) on 35 climatic parameters for the period from 1960 - 2000 from publicly availableWorldClim data. Click here for file

Additional file 4 **Inference of a recent climate driven range expansion in *R. balthica***. Analysis of the freshwater benthos long term monitoring data of the Swedish national monitoring databases at the Swedish University of Agricultural Sciences SLU with canonical correspondence analysis. Click here for file
