# Supplementary material for: Factors and processes shaping the population structure and distribution of genetic variation across the species range of the freshwater snail radix balthica (Pulmonata, Basommatophora)
Source: BMC Evol Biol. 2011 May 20;11:135. doi: 10.1186/1471-2148-11-135 (PMC3115865; doi:10.1186/1471-2148-11-135)

Additional File 1

Species distribution of *Radix balthica* inferred by DNA barcoding

Fig.A1. Distribution of *Radix* MOTU as defined in Pfenninger et al. 2006. South of the Alps, a new MOTU (pink squares) was identified.

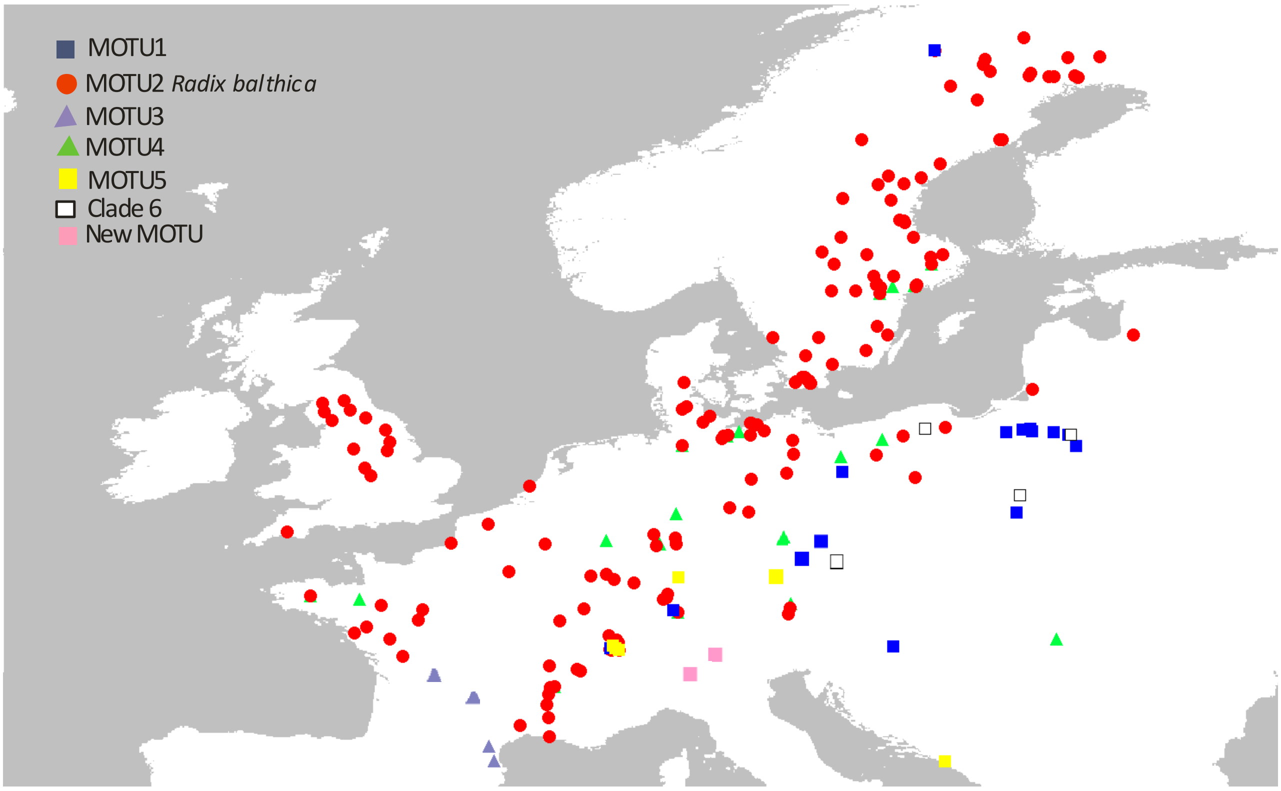

Supplement: Additional file 1 — Distribution of Radix taxa. Spatial distribution of the Radix MOTU as defined in Pfenninger et al. 2006 plus an additional, newly discovered taxon. This map is the basis for the inference of the species range of R. balthica. [file 1471-2148-11-135-S1.PDF]
