# Supplementary material for: Factors and processes shaping the population structure and distribution of genetic variation across the species range of the freshwater snail radix balthica (Pulmonata, Basommatophora)
Source: BMC Evol Biol. 2011 May 20;11:135. doi: 10.1186/1471-2148-11-135 (PMC3115865; doi:10.1186/1471-2148-11-135)

Additional File 2

Sampling site table and spatial distribution of diversity indices, selfing estimates and inferred population bottlenecks

Table A1. Sampling site code, geographical position in decimal degrees latitude and longitude, number of individuals analysed with microsatellites (N_{nuc}), expected heterozygosity (H_E) and standard deviation across loci, mean rarefied number of alleles per microsatellite locus (A) and their standard deviation, number of individuals analysed for mitochondrial variation (N_{mt}), rarefied number of mitochondrial COI haplotypes (H_{mt}), estimated population selfing coefficient (s) and inference of bottleneck occurrence. Populations with no data reported refer to sites from which less than 5 individuals were barcoded and which were therefore not used in analyses. These site were nevertheless used to infer the species range.

Population code	Latitude	Longitude	N_{nuc}	H_E	s.d.	A	s.d.	N_{mit}	H_{mt}	s	Bottle-neck
ABO	47.772	5.985	27	0.23	0.20	2.47	1.00	7	2.77	0.00	No
ALG	61.105	16.819	-	-	-	-	-	5	1.92	-	
ALL	54.896	-2.259	-	-	-	-	-	5	1.00	-	
ATH	54.320	-1.512	-	-	-	-	-	12	4.36	-	
AUG	46.615	7.181	8	0.41	0.28	2.47	1.00	9	2.77	0.74	
BAL	48.273	8.860	12	0.40	0.22	2.47	0.71	9	7.22	0.22	
BER	50.673	2.704	26	0.60	0.15	4.49	1.32	-	-	0.14	
BJE	58.855	17.373	7	0.26	0.27	1.73	0.99	-	-	0.20	
BJO	59.159	16.619	18	0.56	0.25	3.79	1.41	-	-	0.00	No
BOD2	47.667	9.213	-	-	-	-	-	15	6.53	-	
BTB	48.793	7.022	17	0.47	0.28	2.59	1.11	-	-	0.00	Yes
BUX	53.258	-1.905	-	-	-	-	-	5	3.58	-	
CAZ	43.769	3.798	-	-	-	-	-	-	-	-	
CEN	46.938	-1.884	18	0.56	0.17	2.71	0.66	-	-	0.00	Yes
CSV	46.497	7.050	28	0.63	0.18	4.93	2.67	235	6.60	0.10	
DAL	54.829	-2.981	-	-	-	-	-	5	1.92	-	
DAR	59.896	15.688	-	-	-	-	-	6	1.92	-	
DGE	50.189	9.109	15	0.34	0.26	2.34	1.22	11	1.92	0.00	No
DIJ	47.357	5.146	16	0.61	0.17	3.19	1.09	5	4.36	0.00	Yes
DMA	52.207	11.727	29	0.54	0.2	3.90	1.32	11	7.22	0.06	
DPL	52.433	12.941	29	0.55	0.19	4.83	2.06	5	4.36	0.43	
DRB	53.359	9.347	-	-	-	-	-	8	6.53	-	
DRY	52.319	-1.318	-	-	-	-	-	5	2.77	-	
DSL	53.602	10.715	20	0.51	0.21	2.71	1.09	-	-	0.46	
DSM	53.690	10.885	10	0.20	0.22	1.49	0.50	-	-	0.00	No
DSP	54.365	10.316	27	0.48	0.19	4.25	1.65	-	-	0.00	No
DSR	53.698	10.769	11	0.47	0.22	2.59	0.99	-	-	0.14	
EDA	59.834	17.878	8	0.45	0.19	2.35	1.05	-	-	0.00	Yes
EST	46.856	6.840	3	0.00	-	-	-	7	2.77	-	
FBE	44.829	4.820	16	0.66	0.2	6.14	4.09	12	8.56	0.19	
FBO	45.644	5.876	-	-	-	-	-	-	-	-	
FLE	54.699	9.505	12	0.40	0.26	2.34	1.22	-	-	0.58	

FRO	44.053	4.784	12	0.54	0.26	3.89	1.87	12	3.58	0.25	
FTO	45.076	4.822	15	0.96	0.07	7.13	1.94	5	4.36	0.24	
FVI	44.482	4.697	12	0.62	0.14	3.66	1.39	9	5.82	0.46	
FYE	45.706	5.753	16	0.87	0.11	6.66	2.50	5	3.58	0.34	
FYS	59.574	17.914	19	0.54	0.15	3.31	0.86	-	-	0.00	No
GAI	53.195	-0.769	36	0.58	0.23	5.65	1.90	14	5.82	0.15	
GUE	55.539	9.418	-	-	-	-	-	-	-	-	
GVA	46.554	7.080	-	-	-	-	-	-	-	-	
GVD2	46.554	7.074	-	-	-	-	-	-	-	-	
HER	45.117	4.974	-	-	-	-	-	-	-	-	
HOS	59.922	18.310	12	0.39	0.27	2.34	0.97	8	5.82	0.547	
KBW	54.149	11.725	-	-	-	-	-	-	-	-	
KIR	54.214	-2.652	23	0.69	0.25	5.61	2.93	7	3.58	0.17	
LAC	45.976	4.637	-	-	-	-	-	6	4.36	-	
LAV	45.830	4.804	-	-	-	-	-	5	1.92	-	
LEB	62.596	16.437	10	0.50	0.23	2.94	2.71	-	-	0.00	Yes
LIT	55.299	21.387	22	0.27	0.15	2.59	0.99	-	-	0.00	No
LJO	60.004	14.156	-	-	-	-	-	5	1.00	-	
MAR	46.740	-0.658	-	-	-	-	-	13	5.10	-	
MBK	50.007	9.156	18	0.39	0.18	2.47	1.00	13	1.92	0.00	No
MBV	46.492	7.047	-	-	-	-	-	-	-	-	
MEL	53.887	-0.828	22	0.43	0.23	2.70	1.20	10	2.77	0.00	Yes
MOT	58.664	15.310	-	-	-	-	-	-	-	-	
NOR	58.897	16.036	9	0.41	0.20	2.35	0.70	-	-	0.00	No
OBI	48.950	6.756	17	0.52	0.21	3.18	1.3	8	4.36	0.26	
OST	58.884	16.010	15	0.33	0.27	1.97	1.23	6	4.36	0.66	
OUM	63.834	20.337	-	-	-	-	-	-	-	-	
PAT	54.513	-2.924	-	-	-	-	-	5	1.92	-	
PLA	53.979	18.389	10	0.89	0.09	5.88	1.62	-	-	0.01	
PLB	52.279	17.352	30	0.27	0.26	2.58	1.58	-	-	0.00	No
RAN	62.337	16.973	-	-	-	-	-	6	1.92	-	
REM	47.374	-0.301	16	0.39	0.23	2.46	1.12	8	4.36	0.21	
REN	46.385	6.895	19	0.48	0.22	3.06	1.36	-	-	0.00	No
RET	46.360	7.199	16	0.46	0.28	2.70	1.48	-	-	0.00	Yes
RIE	54.149	11.689	-	-	-	-	-	-	-	-	
ROC	46.364	6.938	-	-	-	-	-	-	-	-	
ROS	46.720	7.109	-	-	-	-	-	6	3.58	-	
SAE	66.884	18.023	18	0.32	0.2	2.95	1.00	-	-	0.00	No
SAR	66.885	18.023	11	0.43	0.14	2.83	0.6	5	1.92	0.00	No
SCH	58.873	17.412	9	0.56	0.19	3.55	0.86	7	2.77	0.22	
SDF	60.516	14.808	10	0.38	0.16	2.23	0.43	-	-	0.00	No
SEE	55.434	13.732	-	-	-	-	-	-	-	-	
SEM	57.155	16.419	7	0.59	0.26	3.30	1.41	14	6.53	0.00	No
SHU	66.014	21.949	11	0.13	0.18	1.37	0.48	5	1.00	0.93	
SIF	66.003	22.103	16	0.11	0.20	1.61	0.99	-	-	0.72	
SJO	66.605	19.761	18	0.36	0.25	3.18	1.48	-	-	0.00	No
SJT	65.205	19.489	13	0.19	0.14	1.98	0.87	-	-	0.75	
SKE	57.081	12.468	20	0.54	0.11	2.83	1.17	-	-	0.41	
SKI	67.340	21.092	23	0.21	0.23	2.34	1.22	13	6.53	0.40	
SKJ	66.181	19.931	12	0.07	0.09	1.37	0.48	5	2.77	0.00	No
SKR	55.491	13.751	18	0.45	0.26	2.70	1.39	21	11.77	0.00	No

SKT	66.037	22.828	10	0.39	0.26	2.47	1	-	-	0.00	No
SMA	61.006	16.986	24	0.35	0.22	2.70	1.48	6	4.36	0.00	No
SMB	56.450	13.583	18	0.26	0.20	2.59	0.99	13	5.82	0.66	
SNA	55.582	13.699	20	0.48	0.24	2.94	1.41	7	5.10	0.03	
SNO	58.591	16.143	10	0.55	0.20	3.43	1.12	6	2.77	0.00	No
SNY	58.809	17.368	35	0.40	0.23	2.83	0.93	8	2.77	0.23	
SOV	55.493	13.783	24	0.61	0.24	3.88	2.35	6	3.58	0.10	
SPR	50.019	1.414	44	0.47	0.19	4.14	1.30	-	-	0.33	
SRJ	66.701	23.688	12	0.36	0.22	2.10	0.78	15	1.92	0.48	
SRU	63.834	15.520	14	0.17	0.19	1.49	0.50	-	-	0.23	
SSA	66.123	21.333	22	0.31	0.17	2.35	0.70	-	-	0.53	
SSK	66.028	21.256	9	0.10	0.15	1.37	0.48	-	-	1.00	
SSL	65.985	22.939	14	0.35	0.27	2.46	1.23	-	-	0.63	
SSN	55.566	13.717	39	0.56	0.22	4.70	2.47	19	7.90	0.08	
SSO	55.588	13.669	58	0.71	0.15	7.73	3.87	10	4.36	0.00	No
SSV	58.779	16.178	19	0.39	0.27	2.56	2.12	12	4.36	0.00	Yes
STJ	66.428	19.683	27	0.37	0.20	3.18	1.48	11	2.77	0.37	
SUL	63.010	18.223	25	0.47	0.19	4.25	1.65	12	6.53	0.00	No
SVA	66.674	22.591	11	0.39	0.19	2.10	0.60	-	-	0.00	No
SVE	62.538	17.561	27	0.35	0.25	3.48	3.35	18	4.36	0.00	No
SVO	55.704	13.558	14	0.67	0.15	4.37	1.50	11	7.90	0.13	
SVU	65.670	18.569	12	0.40	0.19	2.35	0.86	-	-	0.34	
SWA	52.591	-1.548	17	0.60	0.20	3.79	0.78	8	2.77	0.46	
SYD	55.552	13.261	18	0.59	0.17	4.94	2.37	12	8.56	0.17	
SYD	55.535	13.245	10	0.40	0.25	2.82	1.62	-	-	0.30	
THO	47.743	0.452	-	-	-	-	-	6	2.77	-	
TON	61.072	16.967	10	0.38	0.29	3.03	1.22	-	-	0.32	
UKP	50.402	-4.203	10	0.48	0.18	2.47	0.87	5	2.77	0.00	Yes
UKY	52.452	0.633	-	-	-	-	-	-	-	-	
VID	57.067	14.042	12	0.53	0.34	4.10	2.63	5	1.92	0.09	
VIK	62.295	16.090	-	-	-	-	-	5	1.92	-	
VOM	55.698	13.554	-	-	-	-	-	5	1.92	-	
N_{pop} = 115			N_{pop} = 81					N_{pop} = 66			N_{pop} = 34
			Σ 1457					Σ 798			
mean	55.18	11.14	17.77	0.44	0.21	3.21	1.38	9.06	4.09	0.20	
s.d.	6.82	7.34	9.00	0.18	0.05	1.31	0.74	5.07	2.19	0.25	

Figure A1. Spatial distribution of expected heterozygosity

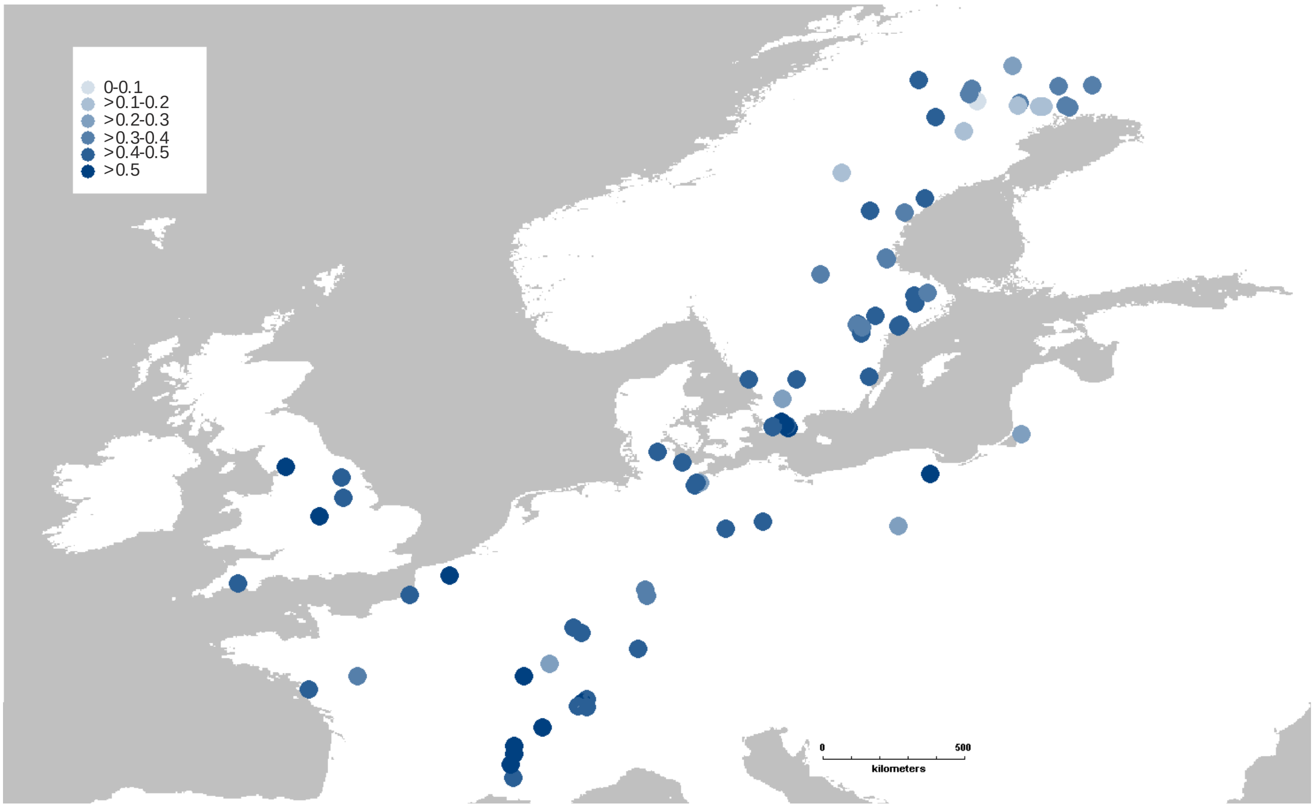

Figure A2. Spatial distribution of rarefied mitochondrial haplotype number

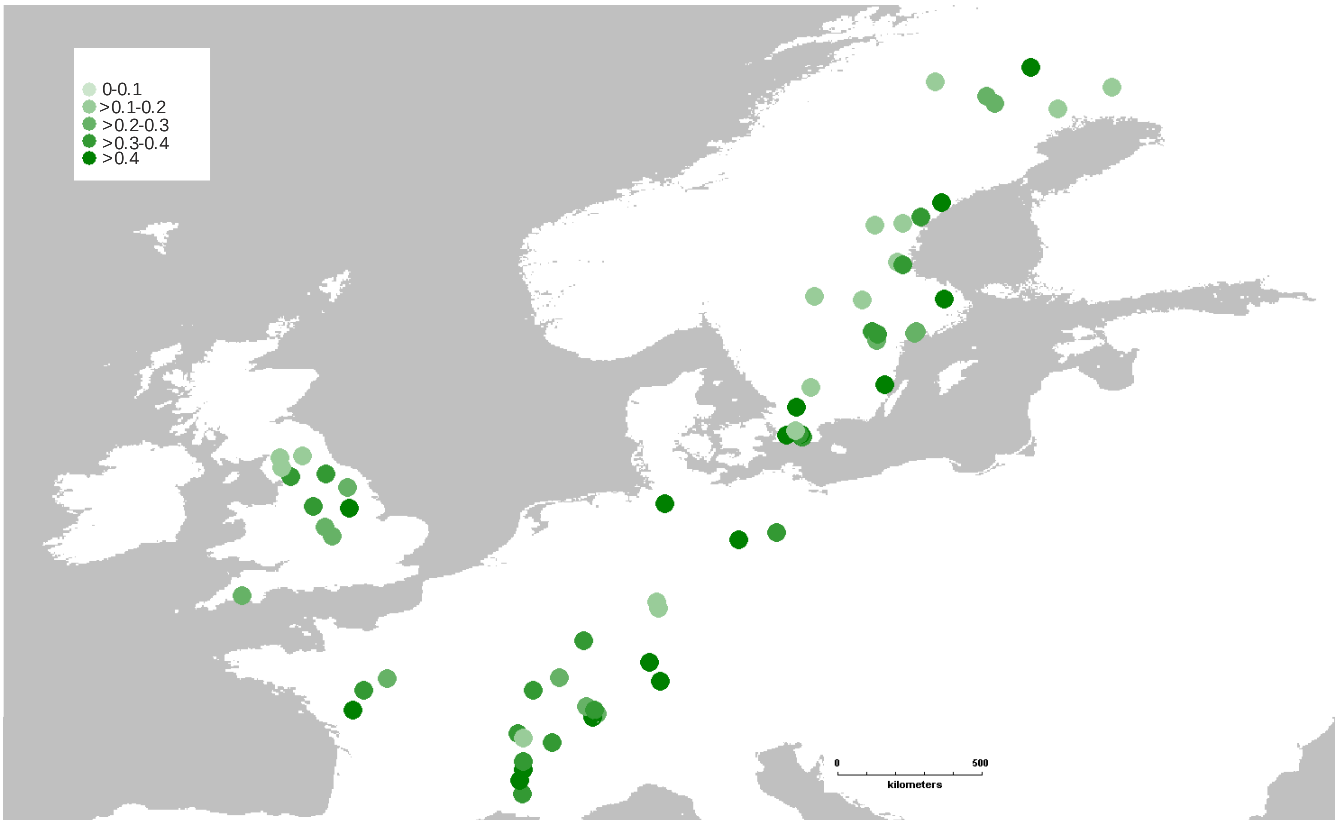

Figure A3. Spatial distribution of population selfing estimates

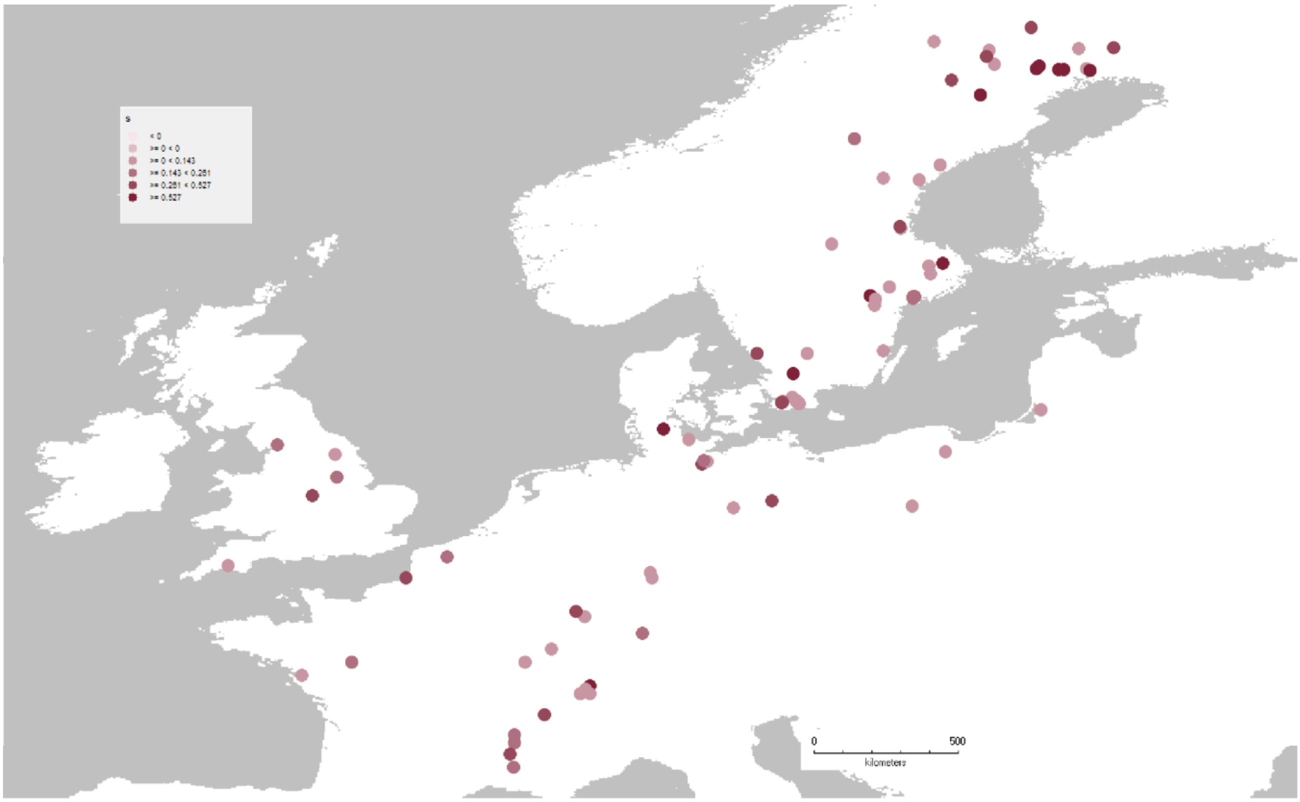

Figure A4. Spatial distribution of populations with recent bottlenecks (orange squares).

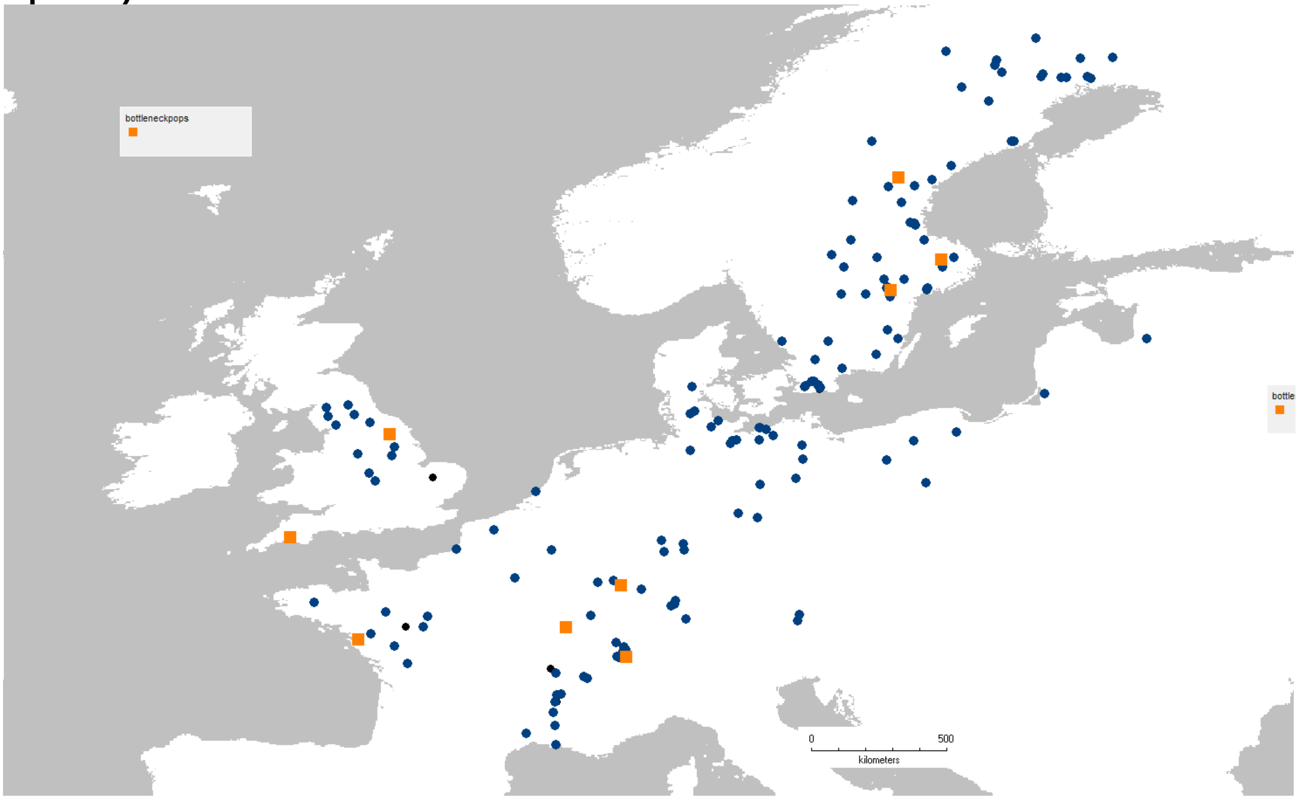

Supplement: Additional file 2 — Sampling site table and spatial distribution of diversity indices, selfing estimates and inferred population bottlenecks for R. balthica. Table of sampling site code, geographical position in decimal degrees latitude and longitude, number of individuals analysed with microsatellites (Nnuc), expected heterozygosity (HE) and standard deviation across loci, mean rarefied number of alleles per microsatellite locus (A) and their standard deviation, number of individuals analysed for mitochondrial variation (Nmt), rarefied number of mitochondrial COI haplotypes (Hmt), number of individuals measured for body size (Nsize). Figures A1 - A3 show a graphical representation of the spatial distribution of He, Hmt and, s, respectively. [file 1471-2148-11-135-S2.PDF]
