# Supplementary material for: Factors and processes shaping the population structure and distribution of genetic variation across the species range of the freshwater snail radix balthica (Pulmonata, Basommatophora)
Source: BMC Evol Biol. 2011 May 20;11:135. doi: 10.1186/1471-2148-11-135 (PMC3115865; doi:10.1186/1471-2148-11-135)
Supplement: Additional file 3 — Assessment of environmental marginality. PCA (principle component analysis) on 35 climatic parameters for the period from 1960 - 2000 from publicly availableWorldClim data. [file 1471-2148-11-135-S3.PDF]

Additional file 3

Assessment of environmental marginality

Figure A1. Results of the PCA (principle component analysis) of 35 climatic parameters for the period from 1960 - 2000from publicly available WorldClim data retained two meaningful (larger than expected eigenvalues according to a broken-stick model) dimensions, accounting for 80.99% of the overall variance. PCA axis 1, a temperature gradient, accounted for 55.83%, PCA axis 2, a precipitation gradient accounted for 25.16% of the total variation. Bars show frequency distribution of populations along the axis.

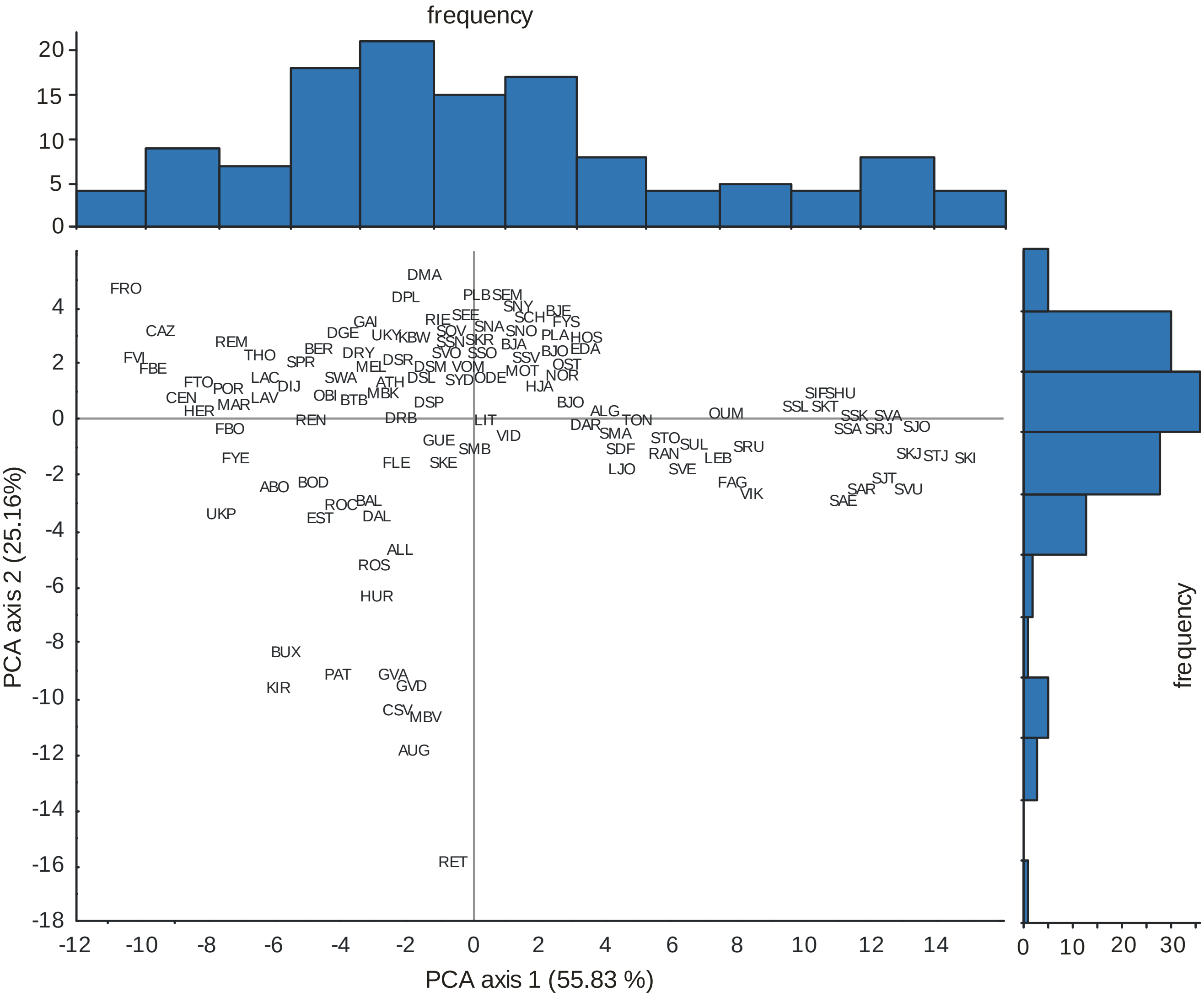

Table A1. Variable-factor correlations for above PCA.

Climate variable	Correlation PCA axis 1	Correlation PCA axis 2
altitude	0.07	-0.81
Jan Min Temp	-0.93	0.21
Jan Max Temp	-0.97	0.17
Jan Prec	-0.66	-0.63
Feb Min Temp	-0.95	0.18
Feb Max Temp	-0.98	0.11
Feb Prec	-0.72	-0.60
Mrz Min Temp	-0.97	0.18
Mrz Max Temp	-0.97	0.09
Mrz Prec	-0.75	-0.60
Apr Min Temp	-0.96	0.20
Apr Max Temp	-0.95	0.12
Apr Prec	-0.71	-0.67
May Min Temp	-0.95	0.26
May Max Temp	-0.90	0.19
May Prec	-0.67	-0.63
Jun Min Temp	-0.86	0.42
Jun Max Temp	-0.79	0.27
Jun Prec	-0.35	-0.77
Jul Min Temp	-0.81	0.48
Jul Max Temp	-0.75	0.23
Jul Prec	0.21	-0.70
Aug Min Temp	-0.86	0.42
Aug Max Temp	-0.86	0.23
Aug Prec	0.01	-0.89
Sep Min Temp	-0.93	0.31
Sep Max Temp	-0.95	0.16
Sep Prec	-0.49	-0.66
Oct Min Temp	-0.92	0.30
Oct Max Temp	-0.98	0.16
Oct Prec	-0.70	-0.39
Nov Min Temp	-0.93	0.25
Nov Max Temp	-0.98	0.19
Nov Prec	-0.68	-0.57
Dec Min Temp	-0.92	0.23
Dec Max Temp	-0.96	0.20
Dec Prec	-0.64	-0.64
Annual Mean Temperature	-0.97	0.22
Mean Diurnal Range(Mean(period max-min))	-0.12	-0.25
Isothermality 2/7	-0.86	-0.12
Temperature Seasonality (C of V)	0.87	-0.06
Max Temperature of Warmest Period	-0.76	0.23
Min Temperature of Coldest Period	-0.94	0.20
Temperature Annual Range (5-6)	0.75	-0.12
Mean Temperature of Wettest Quarter	0.08	0.40
Mean Temperature of Driest Quarter	-0.86	0.20

Mean Temperature of Warmest Quarter	-0.86	0.34
Mean Temperature of Coldest Quarter	-0.96	0.18
Annual Precipitation	-0.61	-0.78
Precipitation of Wettest Period	-0.39	-0.80
Precipitation of Driest Period	-0.54	-0.77
Precipitation Seasonality(C of V)	0.64	0.09
Precipitation of Wettest Quarter	-0.43	-0.81
Precipitation of Driest Quarter	-0.65	-0.72
Precipitation of Warmest Quarter	-0.06	-0.84
Precipitation of Coldest Quarter	-0.69	-0.64
