# Supplementary material for: Factors and processes shaping the population structure and distribution of genetic variation across the species range of the freshwater snail radix balthica (Pulmonata, Basommatophora)
Source: BMC Evol Biol. 2011 May 20;11:135. doi: 10.1186/1471-2148-11-135 (PMC3115865; doi:10.1186/1471-2148-11-135)
Supplement: Additional file 4 — Inference of a recent climate driven range expansion in R. balthica. Analysis of the freshwater benthos long term monitoring data of the Swedish national monitoring databases at the Swedish University of Agricultural Sciences SLU with canonical correspondence analysis. [file 1471-2148-11-135-S4.PDF]

Additional file 3

Inference of a recent climate driven range expansion in *Radix balthica*

Materials and methods:

Data

Data were retained from Swedish national monitoring databases at the Swedish University of Agricultural Sciences SLU. These databases comprise long-term data series from freshwater environments (lakes and rivers) including abundances and biomasses of different organism groups and a variety of environmental parameters. Especially the macrozoobenthos is taxonomically highly resolved and reliable. We used the publicy available monitoring data from 111 Swedish lakes to obtain presence/absence data for Radix specimen. For environmental analysis, 13 data sets of the intensively studied reference lakes, 4 data sets of sites within the largest lakes in Sweden and 5 data sets of rivers were used. The reference lakes have been sampled in different depths and data sets are divided in littoral and sublittoral. All data sets spanned a time of minimally ten years and were sampled once a year, the lakes in late summer/autumn (August to October) after the first mixing, the rivers in spring (May to July) after the snowmelt and the associated flood. The large lakes have the largest data sets beginning with 1969 as the first year included, whereas the sampling of rivers and reference lakes began 1987 and 1988 respectively, 2003 was the last year included. The environmental variables temperature, oxygen, pH, Secchi-depth, conductivity, nutrients (different compounds of nitrogen and phosphorus) and total organic carbon completed the data sets.

Limnological variables

As environmental variables we used all parameters described above. To avoid the effects of co-linearity in the environmental variables, they were subjected to a principal component analysis (PCA). The PCA resulted in four meaningful axes (eigenvalues larger than expected from a broken stick model). EnvPCA1 accounted for 37.9% of total variation and was mainly determined by total phosphorus content (correlation to the axis .0.91) and O2 (-0.64). The second most important axis, EnvPCA2, representing 16.0 % of total variation, received the greatest influence from the pH (-0.68) and ammonium (0.68), while EnvPCA3 (11.9%) reflected the conductivity (0.71). The last axis EnvPCA4 (10.1%) was a gradient of total organic carbon (0.68).

Spatial and temporal variables

Spatial and temporal effects on species abundances were accounted for by the variables latitude, longitude and the respective year of the sampling. As species composition is most likely also influenced by the depth of the sampling point and the total size of the lake, these variables were also incorporated.

Climatic variables

The North Atlantic Oscillation (NAO) is a large scale ocean atmospheric oscillation system which represents fluctuations in the pressure difference between the high pressure zone over the Azores and the low pressure zone over Iceland. It influences air temperature and precipitation over large areas of the northern Hemisphere (Hurrell 1996). An index can be calculated which reflects mild and rainy winters (positive values) and dry and cold winters (negative values). The

NAO indices were derived from the homepage of the National Centre for Atmospheric Research (http://www.cgd.ucar.edu/cas/jhurrell/indices.html). They are based on the difference of normalized sea level pressure (SLP) between Lisbon, Portugal (winter index) or Ponta Delgada, Azores and Stykkisholmur/Reykjavik, Iceland since 1865. The SLP anomalies were normalized by division of each seasonal or monthly mean pressure by the long-term mean (1864-1983 for winter index, 1865-1984 for seasonal and monthly indices) standard deviation.

As climate variable we used the NAO winter index as described above. Water temperatures during the winter months were thought to exert the greatest influence on species communities. Unfortunately, measurements from this season were incomplete. Therefore, water temperatures in October, taken in 5 m depth or, if the lake was not that deep, the deepest measurement, were taken as a proxy for the amount of warmth the respective lake had accumulated during summer. This stored caloric energy determines also the temperature development during winter (Brönmark & Hansson 1996). The rare missing data from years with no temperature records were filled with the average of the preceding and the following year (Variable WaterTemp). We tested for the presence of a significant linear trend in the temperature data using the Mann-Kendall Test and calculated Sen's slope estimate with the Excel-application MAKESENS 1.0, provided by the Finnish Meteorological Institute.

In order to extract a trend signal from the year-to-year variation in the temperature data, we applied the caterpillar method as implemented in the software CATERPILLAR 1.0 (Group C 1997). To this end, the time series was centred before decomposition with a lag size of 10. For reconstruction of the trend, only the caterpillar average was used. The resulting temperature trend time series was also tested for significance as described above (Variable TrendTemp).

Data analysis

Canonical correspondence analysis (CCA) (Ter Braak 1986) was applied as multivariate ordination technique to analyse the spatial and temporal variation in species abundance data. To remove the unwarranted effects of rare species on the ordination results, species occurring in less than four samples and/or with less than 100 individuals were excluded from further analysis. This yielded a matrix of 147 species. Abundance data were square-root transformed prior to analysis and rare species downweighted.

Initially, principal patterns in species distributions were ordinated via detrended correspondence analysis (detrended by segments). The gradient length exceeded 3 in standard deviation units, indicating that a unimodal model adequately represents the species' response (Jongman et al. 1995). Statistical correlations between species abundances and environmental variation were further assessed via CCA, a non-linear eigenvector ordination technique in which the axes are constrained to be linear combinations of the measured environmental variation. The environmental variables were ordered by forward selection according to the amount of explained variance in the species data. Treating each variable as the sole predictor variable in a first step, all environmental variables were ranked on the basis of the variance they explained separately, thus representing marginal effects. Selecting the best fitting variable in a second step as covariable, the remaining variables were again ranked according to their explanatory power for the remaining variance. This procedure was repeated until all variation was explained, thus yielding the conditional or unique effects of each variable. At each step, the statistical significance of each variable added to the model was tested using a Monte-Carlo permutation procedure with 999 unrestricted permutations. Statistical significance of the first 4 axes and

associated constrained eigenvalues was also tested with 999 unrestricted permutations, using the samples scores as covariables for the higher order axes (Ter Braak & Verdonschot 1995).

As we were predominantly interested in the effect of the climatic variables on the species composition, we performed a series of partial CCA by considering in turn the spatial, temporal and climatic variables as explaining factors and all remaining variables as cofactors. The partial models were also tested using a Monte Carlo permutation approach. The percentage of species variation explained by the different CCA models was calculated as ratio of the sum of canonical eigenvalues over the total inertia in an unconstrained CA. All ordination calculations were performed with CANOCO 4.5 (Ter Braak & Smilauer 2002).

Results:

Figure A1: Extent of range expansion of *Radix* in Sweden between 1995/96 and 2003/04. Red squares mark the lakes monitored. A) Swedish distribution range 1995/06 enclosed by outline. B) Range 2003/04. Expansion area marked with hatched whitish layer.

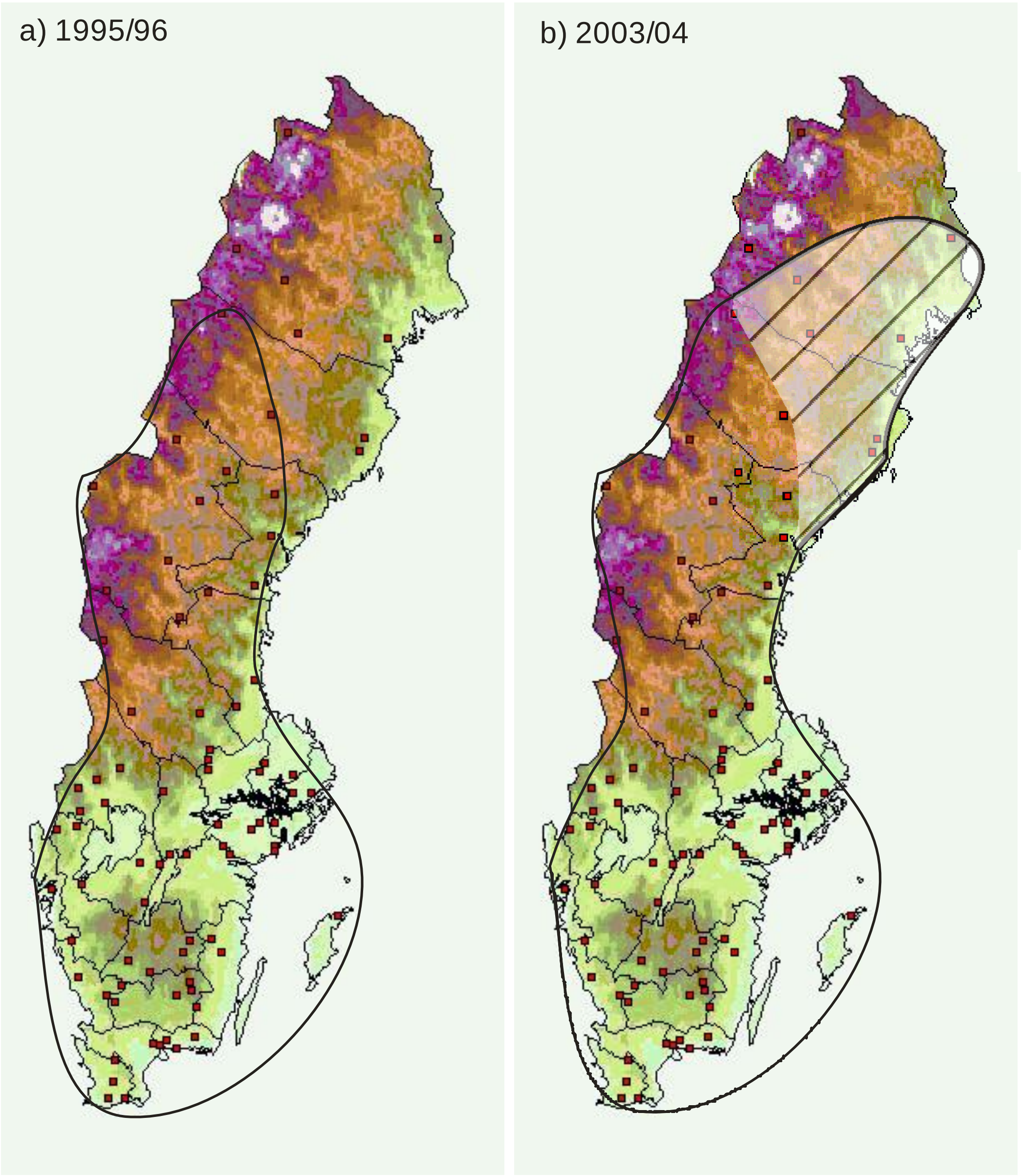

Figure A2: Species ordination results from canonical correspondence analysis (CCA) of gastropod abundance data. The plot shows the species scores along the first and second axes in relation to the environmental variables. The directions and lengths of the arrows indicate importance and correlation to the respective axes. The abundance of *Radix* (in red) is significantly (r = 0.7, p < 0.001) correlated to the (positive) trend in lake temperatures (red arrow).

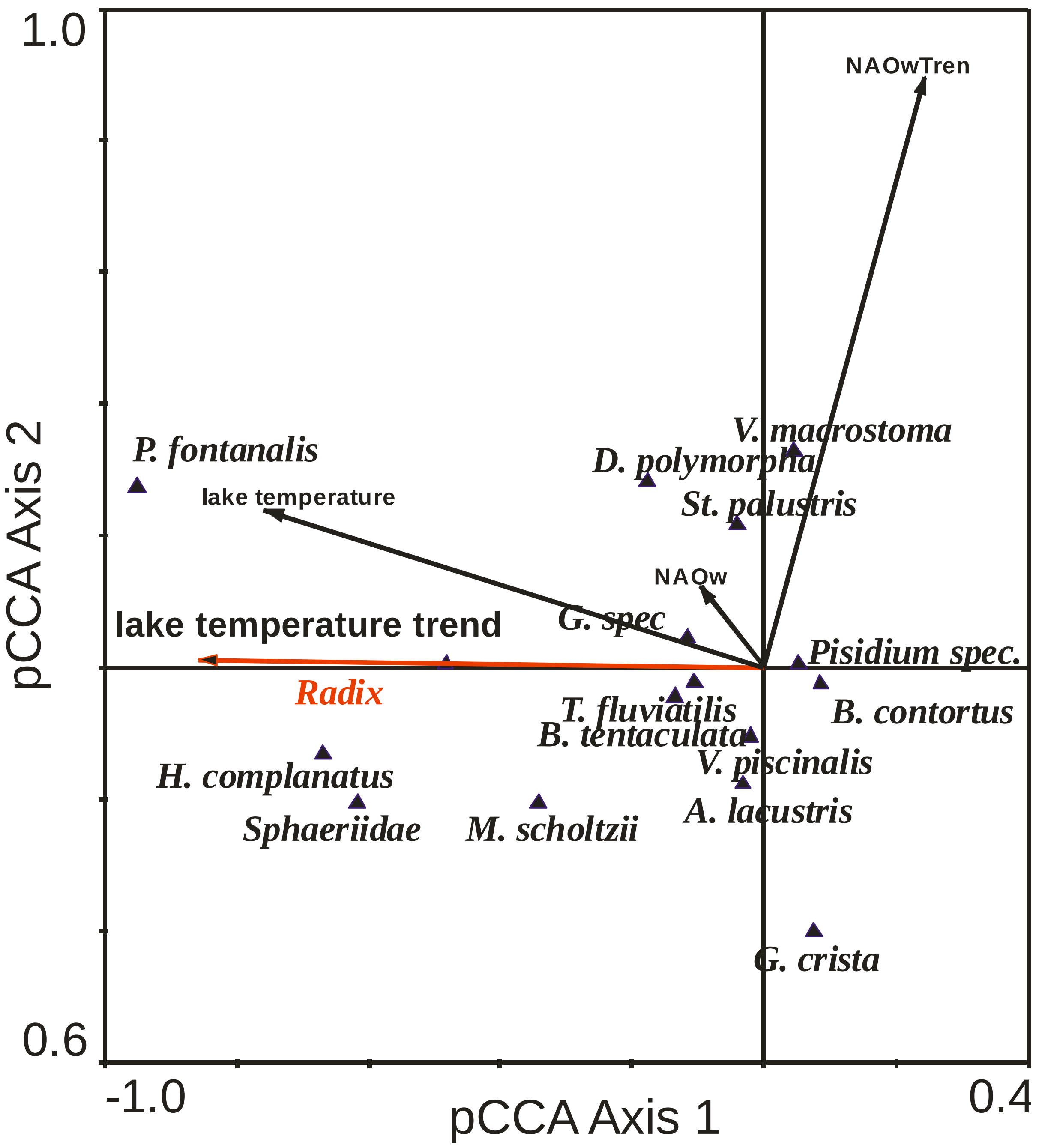

Supplemental References:

Brönmark C, Hansson LA (1996) The Biology of Lakes and Ponds. Oxford University Press, Oxford

Group C (1997) Caterpillar: Program for time series analysis 1.0. St. Petersburg

Hagberg J, Tunberg BG (2000) Studies on the covariation between physical factors and the long-term variation of the marine soft bottom macrofauna in western Sweden. Estuarine, Coastal and Shelf Science 50:373-385

Hurrell JW (1996) Influence of variations in extratropical wintertime teleconnections on Northern Hemisphre temperature. Geophysical Research Letters 23:665-668

Jongman RGH, Ter Braak CJF, Van Tongeren OFR (1995) Data analysis in community and landscape ecology. Cambridge University Press, Cambridge

Ter Braak CJF (1986) Canonical correspondence analysis: a new eigenvector technique for multivariate direct gradient analysis. Ecology 67:1167-1179

Ter Braak CJF, Smilauer P (2002) Canoco for windows Version 4.5. Biometrics - Plant Research International. Wageningen

Ter Braak CJF, Verdonschot PFM (1995) Canonical correspondence analysis and related multivariate methods in aquatic ecology. Aquatic Science 57:255-289
